# A Recent Outbreak of Rötheln

**Published:** 1892-03

**Authors:** William Johnstone Fyffe


THE BRISTOL

Medico-Chirurgical Journal.

MARCH, 1892.

### A RECENT OUTBREAK OF RÖTHELN.

BY

William Johnstone Fyffe, M.D.

During the summer months of 1891 German measles was to some extent prevalent in Clifton. As it is a disease affecting principally young persons, and as it is exceedingly infectious, it was natural to expect that large schools would be visited by it. This was the case in Clifton College during the summer term. In the previous term ending at Easter there were some cases in the Junior School, mainly affecting one house. The short Easter holidays were not sufficient to get rid of the infective possibilities of the disease in the neighbourhood, and consequently it broke out afresh soon after the commencement of the summer term.

It is absolutely impossible to stop an epidemic of measles, either of the ordinary or the German type, in a large school. Both forms are so infectious in their early stages that when one case takes place in a school, most probably several others have been already infected, and so the disease goes on until every unprotected boy has been attacked. Dr. Haig Brown, the medical officer of Charterhouse, states that in 1886 one case of rötheln or German measles was introduced into a boarding-house in the school, and from that case, in spite of every precaution by immediate isolation and disinfection, the epidemic went on until over 200 boys had been attacked. This is the experience of most large schools.

Before recording the details of this outbreak, which presented some features of a remarkable and perplexing character, I may briefly notice the present position of our acquaintance with this disease, which, in addition to its puzzling clinical features, labours under the misfortune of possessing a bewildering nomenclature.

For one disease to be called by no less than seven different appellations is a misfortune, and leads to confusion. Rötheln, rubeola scarlatinosa, rubeola morbillosa, rubeola notha, epidemic roseola, rubella, and German measles form a conglomerate specimen of medical nomenclature which is irritating and misleading both to the profession and to the lay public.

Possibly this may arise from the fact that rötheln (as I shall call it) is comparatively a new disease. It had no place in the *Nomenclature of Diseases* before the year 1885, and yet long before that it had been the subject of comment, and up to the present time there are few diseases about which so much controversy has raged. It is a disease which is seen only in epidemics, and the type and character of these epidemics appear to vary so much that the recorded experience of one observer seems flatly to contradict that of another. Some describe it as false measles; others as an anomalous scarlet fever; and a third class have imagined it to be a hybrid, a disease partaking of both these exanthems, and becoming in this way a kind of pathological monstrosity.

These views, however, have now ceased to exist, and I suppose that at the present time there is a generally-accepted belief that rötheln is a distinct exanthem, giving no protection to the individual who has had it either against the infection of measles or scarlet fever.

I will now give the results of my experience during the prevalence of an epidemic which took place among the boarders of Clifton College during the months of May, June, and July last.

The epidemic began on the 19th May, and, in spite of every precaution taken in the way of immediate isolation, the disease spread from house to house until 91 boys had been attacked. Its manner of progress was very similar to that which I have always observed in epidemics of ordinary measles. That is to say, there was a fresh outbreak of the disease about once in fourteen days, which I think marks the period of incubation pretty fairly, although in some cases the period appeared to be much shorter. By far the majority of the cases assumed the character with which we are all familiar, and which for the sake of order I will briefly recapitulate.

The boy or girl attacked with that mild form of rötheln which was prevalent last summer experiences none of the prodromata of the other exanthems, neither vomiting nor shivering, and little or no *malaise;* possibly there may be a little headache, stiffness in the neck, or a little sore throat. The first thing, as a rule, which attracts the attention of the patient or those about him is the rash. There is a pink mottled rash observed over the chest and back, on the arms, round the neck, and behind the ears.

The spots are separate, slightly raised, but not crescentic. There is often slight swelling of the post-cervical glands. There may be catarrhal swelling in the throat, but no implication of the submaxillary glands. The temperature is seldom above 100° or 101°. This condition lasts from four to six days. The rash fades, all the symptoms disappear, and the whole thing is over. There may be a little branny desquamation, but very little.

Now if this type of the disease had been maintained all through this outbreak in the school, this paper would not have been written. But I had once before observed a departure from this ordinary type of the disease, and I was on the look-out for something of the kind again. The instance to which I refer was this.

At the end of the summer term of 1890 a boy had been under treatment in the sanatorium for rötheln of the type I have just described. He had got quite well apparently; his rash had disappeared. He was the only patient in the sanatorium. He was sent home at the end of a fortnight. Immediately on his arrival at home I received a telegram from his mother, informing me that he had suddenly developed a bright scarlet rash over his body, and asking me if there were any cases of scarlet fever either in the sanatorium or in the school. I replied in the negative. In a few days I received a letter informing me that the physician in attendance had decided that the attack was a recrudescence of rötheln, and that the boy was convalescent, but was desquamating.

This in a more extended and intensified form is the experience I have had with this disease during the last summer term.

Out of the total number of 91 cases which occurred, there were thirteen in which the scarlet rash was developed. In *seven* of these the primary rash had all the characters of the simple pink mottled eruption of rötheln, and the secondary rash, which developed at a period varying from two to six days after the first, was so like that of scarlatina as scarcely to be distinguished from it. In five cases the primary rash was not pink or mottled (at least when I saw it first), but a general uniform red eruption over the body.

I will now give some brief details of these cases. A boy, B., was admitted on the 21st June with the mottled pink rash of rötheln (he had had ordinary measles previously). The rash covered the chest and back; there was a little on the face and behind the ears. He had no fever or sore throat. The rash faded on the third day, and he was allowed to get up. Six days after he had headache, sore throat, a temperature of 101°, and a bright red rash covered the body. The temperature was normal on the third day after the rash appeared, and he never showed the least sign of desquamation.

A boy, L., from another house, came in on the 22nd June with the mottled pink rash, and a previous history of measles. Three days afterwards the red rash appeared over the trunk, arms, and legs. In this case it was very evanescent and faded in about forty-eight hours, and was not attended with any fever, sore throat, or subsequent desquamation.

A boy, V., again from a different house, came in on June 23rd, also with a previous history of measles. He had the mottled pink rash over the chest, back, and abdomen, without any fever. On the fourth day after admission the red rash suddenly developed over the body. The fauces were covered with a similar rash, but there was no swelling of the tonsils. The temperature was

101° on the first day, but fell to the normal point on the third, and never rose again. This boy desquamated a good deal, but it was principally branny in character.

A boy, B. (2), from the same house as the last case (also with a previous history of measles), came in on the 26th June with the pink mottled rash, and without any symptoms of pyrexia. The rash faded on the third day. On the 1st July, five days after admission, he developed a bright red rash over the whole body, and simultaneously his temperature rose to 103°, but steadily fell from day to day till it reached the normal on the seventh day after its appearance. He had a good deal of sore throat, but hardly any swelling of the tonsils and no glandular implication. Desquamation in this case was very marked and prolonged. He was not allowed to leave the sanatorium for six weeks after his admission. This was one of the cases which made diagnosis very difficult; for the symptoms were so identical with those of scarlet fever, that I do not hesitate to say that most medical men, seeing it for the first time, would have pronounced it to be so. But I will discuss this question farther on.

The fifth case, that of E., from a third house, presented symptoms almost identical with those of B. (2). He came on June 29th, with the usual mottled rötheln rash, from which he apparently recovered, and on the sixth day after admission developed a bright scarlet rash, with some vomiting and sore throat. His highest temperature was 101.3°. The rash disappeared on the seventh day. He desquamated a good deal, and was kept for six weeks in the sanatorium.

The sixth case, A., from a fourth house, was also admitted on the 29th June for rötheln similar to the others. The rash faded on the third day. He was then attacked with sore throat and fever, having a temperature of 103° on the first day, with a bright red rash over the body. This faded with the fall of temperature, and had quite disappeared on the sixth day. There was some desquamation, but not so much as in the previous cases. This boy went home at the end of a month from the beginning.

The seventh and last of this class of cases was that of C. (2). He came from a fifth house on the 27th with rötheln. He was not at any time associated with any of the previous cases. He had the mottled rash for three days. He then developed a peculiarly bright red rash over the body, without any sore throat. He had very slight fever, but the constitutional disturbance was at no time commensurate with the intense character of the rash. In fact, the boy did not feel ill at all. He remained fourteen days in bed, because, although the scarlet rash faded about the sixth day, it was succeeded by a densely-scattered eruption of small pustules over both hips and thighs, without any constitutional disturbance whatever except for one day.

Then I may mention, in addition, the case of one of the masters' sons, a boy, C. (3). I mention it because I watched this case very specially, and examined him several times in the day during the first few days of his illness, and I was able to satisfy myself that the separate spots of the pink mottled rash of rötheln gradually and perceptibly coalesced and deepened in colour, until the whole body was covered with a uniform smooth red eruption.

All these cases, with the exception of C. (2), came into the sanatorium within a few days of each other, and were more or less together in the infectious house. They came from *five* different houses. They all began with the ordinary pink rash, and then all developed the scarlet rash within periods varying from three to six days.

There were, however, *five* other cases, which differed from those I have described in this respect, that they did not develop the usual preliminary mottled rash at all; or if it was developed, it lasted such a short time as not to have been noticed either by myself or by any of the nurses or house matrons.

A boy, C. (4), was admitted on July 2nd with a bright red rash over the body. Temperature, 101°; no sore throat. His temperature was normal on the third day, and remained so. The rash faded on the fifth day. On the sixth it reappeared over the face and neck, without any fever, and two days after it appeared profusely on the legs. In this case there was no desquamation whatever.

There were two other cases very similar from the same master's house within a week, and a third, the son of one of the masters, who was under the care of Dr. Rogers[1]. They were very mild in character. In two there was neither sore throat nor desquamation.

But on the 11th July a boy, C. (5), was admitted from a house from which no other case of scarlet rash had come; and I must confess it was a case which puzzled me very much, and about which I was unable for some time to make up my mind on the question of diagnosis.

When I was sent for to see this boy at his master's

[1] Dr. Rogers has furnished me with the following facts: The patient whom he attended was admitted to the sanatorium on the 12th July with the scarlet rash already described; on the 14th the rash had nearly gone and his temperature was normal, and the sore throat had disappeared. Some fine branny desquamation began on the 26th, and spread slowly over the body and limbs, and was well marked on the hands. At no time was there any albuminuria, or was the boy in the least degree unwell.

house, I found him apparently very ill. He had severe sore throat, a temperature of 103°, and a bright red rash over the entire body. He was removed to the infectious part of the sanatorium at once. The case had all the appearance of scarlet fever. As soon, however, as the rash became fully developed he began to get better; he never had an anxious symptom. He was kept for six weeks in the sanatorium, and desquamated a good deal. But now comes the puzzling part of the case. I looked into the previous medical history of this boy. I found a record of his having had scarlet fever three years before. I made careful inquiry of his parents, and was informed by them that this was perfectly true, and they stated, moreover, that the attack was followed by profuse and prolonged desquamation. This was the last case of this red rash which occurred.

This is then a brief detail of certain facts occurring in this outbreak: a total number of 91 cases, and out of that 13 special cases, 8 of whom developed a secondary scarlet rash, succeeding a preliminary pink mottled rash, and 5 exhibiting the scarlet rash from the beginning, without the preliminary pink rash at all.

I will now make a few criticisms on these special cases. The question which naturally occurred to my mind when these cases of scarlet rash began to appear was this: Was this a new departure, a new feature in the history of rötheln, or was it possible that a mild case of true scarlet fever had in some way got into the sanatorium and had infected the others?

But then the difficulty arose of accounting for cases which had not presented any preliminary pink rash, but who developed the scarlet rash in their masters' houses and not in the sanatorium. And there was the case of

C. (5), whose symptoms were so like those of scarlet fever, and yet with the clearest evidence that he had had severe scarlet fever three years before.

I need not say that under these circumstances I availed myself of the opinion and assistance of some of my medical friends. I asked my friend, Dr. Fox, to see some of the earlier cases. I forget at this moment if he gave a positive opinion one way or the other, but I know that, observing the desquamation which was going on in some of them, he strongly counselled great precaution about sending the boys home.

Then if these cases were undoubtedly scarlet fever, I was of course bound to report them to the Medical Officer of Health. So I asked Dr. Davies to look at them, which he very kindly did, and he gave it as his opinion decidedly that they were not cases of scarlet fever. Then my friend, Dr. Harrison, who is so experienced in cutaneous disease, very kindly also came to see the boys, and he agreed with Dr. Davies that these were instances of rötheln almost indistinguishable from scarlet fever.

Dr. Rogers, to whose case I have referred, also agreed in opinion with Drs. Davies and Harrison. Finally, I arrived at the conclusion myself that I was not dealing with scarlet fever, but with rötheln—or that form of it which has been called rubeola scarlatinosa—and for the following reasons:

(1) There were no cases of scarlet fever in the school or in the neighbourhood. Rötheln, and that alone, was prevalent; and I think it very unlikely that the infection of scarlet fever could have gained an entrance into the sanatorium, isolated as it is.

(2) The clinical features of the cases presented aspects different, when analysed and observed for some time, from those of scarlet fever. The prodromata, whether in the cases of secondary rash or in those where the red rash was not preceded by the pink mottled rash, were so very short as not to be noticeable.

(3) The throat symptoms were not nearly so severe as one would have expected to have found them where the rash was so prominent a symptom. There was general redness of the fauces, but very little swelling of the tonsils.

(4) In not one of these cases could I say for certain that they presented the usual characteristic strawberry tongue of scarlatina. The tongue was mostly covered with white fur, with slightly red tips and edges, but there was no special prominence of the papillæ.

(5) There was no glandular implication. There was some tenderness of the post-cervical glands behind the sterno-mastoid. This has been considered to be the distinguishing feature of rötheln. By some it is regarded as pathognomonic. I should hardly be disposed to go so far as that; but at any rate there was no such glandular implication as might have been expected in such cases had they been scarlet fever.

(6) I think also there were some distinguishing points in the rash itself. It was bright scarlet certainly, but it had more of the uniform velvety look of erythema than the punctate rash of scarlatina.

(7) Another strong point of difference appeared to me to be the entire want of all proportion between the intensity of the cutaneous rash and the general condition of the patients. The case of C. (2) for example, where the rash, beginning as a pale pink mottled eruption without any pyrexia whatever, developed so rapidly that in a single night the whole body was bright red, and yet with very little constitutional disturbance; and in most of the cases, when the rash was fully developed, the symptoms began to abate. This is not the usual behaviour of scarlet fever. As a rule, the trouble begins with the rash.

(8) In not one case throughout was there a single instance of any kidney complication. In every one of these 13 cases the urine was examined daily, and no trace of albumin was ever discovered.

(9) Lastly, several of the milder cases, where there had been a slight red rash with some branny desquamation, were sent home at the end of three weeks. In not one instance was any other member of their families attacked.

But on the other hand, there was one feature in these cases which made me hesitate for some time before pronouncing a decided opinion that they were *not* scarlatina cases. That was, the unmistakable desquamation which took place in several of them, I may say, in all of those in which the pink rash was followed by the red rash. It was more than branny desquamation, it was flaky, and it was well marked on the palms and soles of the feet. But there were some cases of the red rash in which there was no peeling at all, and this leads me to the opinion that the amount of desquamation is not dependent upon the intensity of the rash even in scarlet fever. I had a boy under my care later on who had a sore throat. His house matron said she thought he had a rash on his chest the night before I saw him, but I could not see it. However, as a precautionary measure, I took him to the infectious part of the sanatorium, and he desquamated like the worst case of scarlet fever I ever saw.

I have now stated the facts respecting this outbreak and the reasons which led me to conclude that these cases were instances of rötheln, or, if the name is more suitable, of rubeola scarlatinosa.

I find a record in the *British Medical Journal* of 1887, Vol. I., page 826, of two epidemics at Charterhouse School, published by the medical officer, Dr. Haig Brown. One occurred in 1886, when there were 202 cases. There was no resemblance to the rash of scarlatina among any of the cases in this outbreak; but in the year 1884, 13 cases of infectious disease took place in which the rash exactly resembled that of scarlatina, and there was consecutive peeling, but no nephritis. But the most important part was, that five out of the 13 had previously had scarlatina, and that not one of these contracted rötheln in the epidemic in 1886, although nine were still in the school and were exposed to infection like the other boys. Dr. Brown concludes that there are two forms of the disease, or rather two varieties of rash—one scarlatinal, the other rubeoloid.

Dr. Liveing, in a clinical lecture on Rötheln, delivered in 1874, states that the premonitory fever is generally mild: but in some of Dr. Haig Brown's cases the temperature reached 105°; and Dr. Fox, in his observations on Temperature, published in the *Medical Times* of April, 1870, states that in the first two days of rötheln the temperature rises to 104°. Dr. Liveing confirms the experience I have described as to the coalescing of the pink measly rash, the uniting of the patches, and the development of a uniform red colour over the entire body resembling scarlatina, and that its disappearance is followed by desquamation and sometimes by albuminuria. But with regard to the development of the secondary rash which I have described, Dr. Liveing makes this pertinent remark, that "this disease perhaps accounts for some supposed errors of diagnosis, when a patient, said by the first practitioner who has seen him to be suffering from measles, is pronounced by a second under whose notice he comes at a later period to be suffering from scarlatina."

I must not take up further space in discussing the question. In such cases the diagnosis is difficult, and it is best to be on the safe side and to take such precautions as may be necessary until desquamation has entirely ceased.

